# *Planococcus dechangensis* NEAU-ST10-9^T^ Promotes Maize Seedling Root Development: Evidence from Effective Fluorescence Tracking

**DOI:** 10.3390/microorganisms14051139

**Published:** 2026-05-17

**Authors:** Qi Zhou, Zhenyu Huang, Han Li, Jiaying Xiong, Meixia Chen, Yan Liu, Wei Liu, Yanlai Yao, Ramon Gonzalez, Yu Li, Aiqin Shi, Fuping Lu

**Affiliations:** 1 Key Laboratory of Industrial Fermentation Microbiology of the Ministry of Education, Tianjin Key Laboratory of Industrial Microbiology, National Engineering Laboratory for Industrial Enzymes, College of Biotechnology, Tianjin University of Science and Technology, Tianjin 300457, China; 2 Xianghu Laboratory, Hangzhou 311200, China; 3 Mojia Biotech PTE. Ltd., 6 Raffles Quay #14, Singapore 048580, Singapore

**Keywords:** *P. dechangensis* NEAU-ST10-9^T^, conjugation system, fluorescence tracking system, rhizosphere colonization, plant growth-promoting microorganism

## Abstract

Understanding the interaction between plants and rhizosphere microorganisms is critical for the development of biofertilizers. Fluorescent labeling of rhizosphere microorganisms serves as a key strategy to track their behavior during plant–microbe coculture. However, most newly isolated strains are novel and lack available molecular tools for such studies. In this research, *Planococcus dechangensis* NEAU-ST10-9^T^ (*P. dechangensis* NEAU-ST10-9^T^), a salt-tolerant strain, was obtained from the China General Microbiological Culture Collection Center (CGMCC). It significantly increased maize root length by approximately 1.56-fold. To investigate the underlying mechanism, a donor strain (*Ec102*) and a shuttle plasmid (pAS104) were engineered to mediate conjugation with *P. dechangensis* NEAU-ST10-9^T^ and drive GFP overexpression in the bacterium, generating the genetically labeled strain *Pd103*. The fluorescence intensity (expressed as GFP/OD_600_, arbitrary units) of *Pd103* increased with bacterial growth and was approximately tenfold higher than that of the wild-type strain after 16 h of culture. Following inoculation onto maize seeds, confocal microscopy analysis revealed that *Pd103* colonized the epidermis and endodermis of maize roots. These results indicated that *P. dechangensis* NEAU-ST10-9^T^ could invade maize roots and promote maize seedling growth. In summary, we have successfully established a robust fluorescence labeling and tracking system tailored for *P. dechangensis* NEAU-ST10-9^T^, which constitutes a valuable tool for elucidating the cellular and molecular mechanisms governing its plant–microbe interaction.

## 1. Introduction

*Planococcus* species are Gram-positive or Gram-variable bacteria commonly found in diverse environments such as soil and seawater. Some species function as plant growth-promoting rhizobacteria (PGPR), enhancing plant growth. For example, *Planococcus* soli WZYH02 improves maize growth, biomass yield, and antioxidant levels under salt stress, though the underlying mechanism remains unclear [1].

To understand interactions between microorganisms and plants, an effective approach is to label microorganisms with fluorescent markers and track them during coculture with plants [2,3]. Green fluorescent protein (GFP) is commonly used for this purpose [4]. However, there are no reports on labeling *Planococcus* species with fluorescent protein. This is probably because, in naturally occurring *Planococcus* species, it is difficult to acquire plasmids with classical methods such as chemical transformation and electroporation.

Conjugation is a contact-dependent horizontal gene transfer mechanism that enables genetic material transfer from one bacterium (the donor) to another (the recipient) [5]. The conjugative machinery comprises conjugative pilus proteins, the Type IV secretion system (T4SS) and the relaxosome components [6]. Transferred DNA must contain an origin of transfer (oriT), which is recognized and cleaved by relaxase in the donor strain [7]. Key requirements for successful conjugation include a donor strain, a recipient and an effective method to separate donors from recipients after conjugation [8]. Several organisms can serve as donors, including *E. coli*, *Mycobacteria smegmatis* and hyperthermophilic *Archaea*, among others [6,9,10]. To expand the applicability of donor strains, researchers have engineered them to be auxotrophic, allowing differentiation from recipients using nutrients as selection markers [11].

*P. dechangensis* NEAU-ST10-9^T^ is a model strain of *Planococcus* species isolated from saline-alkaline soils in Dechang Township, Zhaodong City, China [12]. Given the importance of GFP labeling for *Planococcus* species, the present study developed an auxotrophic donor strain and a shuttle plasmid to achieve high-efficiency GFP expression in *P. dechangensis* NEAU-ST10-9^T^, and demonstrated that *P. dechangensis* NEAU-ST10-9^T^ promoted maize root growth by colonizing its root epidermis and endodermis.

## 2. Materials and Methods

### 2.1. Strains, Media and Growth Conditions

The strains and plasmids used in this study are listed in Table 1. During strain construction, cultures were grown aerobically at 30, 37, or 39 °C in Luria broth (composition per liter: 10 g Difco tryptone, 5 g Difco yeast extract, and 5 g NaCl). Ampicillin (100 mg/L) and chloramphenicol (34 mg/L) were added as required.

### 2.2. Plasmids Construction

Cloning of *ori*T into pNW33n-GFP: For construction of pAS101, the *ori*T DNA fragment from the RP4 plasmid was synthesized by Tsingke Biotechnology Co., Ltd. (Beijing, China) and cloned into pUC18. The *oriT* element from pAS101 was amplified using primers *ori*T-F/*ori*T-R, yielding Fragment I. pNW33n-GFP was digested by *Eco*RI (New England Biolabs, Ipswich, MA, USA), and used as a template to amplify Fragment II with primers pNW33N-F/pNW33N-R. Fragment I was then cloned into Fragment II using the Vazyme ClonExpress Ultra One Step Cloning Kit (C115-01) to generate plasmid pAS102.

Cloning of the minimal replicon from pCZ1 into pAS102: The minimal replicon of pCZ1 was first synthesized by Tsingke Biotechnology Co., Ltd. and cloned into pUC18 to create pAS103. After digestion with *Hind*III-HF^®^ (New England Biolabs), pAS103 served as a PCR template to amplify Fragment I (containing the pCZ1 minimal replicon) using primers rep-pPCZ1-F/rep-pPCZ1-R. pAS102 was digested with *Bsp*HI (New England Biolabs) and was amplified with primers pNW33N-origin-cloning-F/pNW33N-origin-cloning-R to produce Fragment II. Fragment I was cloned into fragment II using the Vazyme ClonExpress Ultra One Step Cloning Kit (C115-01) to generate plasmid pAS104.

### 2.3. Gene Modification

To delete *dapA* in *E. coli* S17-1, a two-step recombination method was employed without leaving any foreign DNA as described previously [13]. For the first step, *cat-sacB* cassette was amplified using primers *dapA*-*cat*-F/*dapA*-*sacB*-R with AS010 as the template. Both primers *dapA-cat*-F and *dapA-sacB*-R contained 50 bp of homology to the *dapA* gene. This fragment was introduced into *E. coli* S17-1 (pKD46) by electroporation, and colonies were selected on LB plates supplemented with DAP, ampicillin, and chloramphenicol, yielding strain *Ec101*.

The upstream homology fragment was amplified using primers *dapA*-up/*dapA*-1 with the *E. coli* S17-1 genome as a template, while the downstream homology fragment was amplified using primers *dapA*-2/*dapA*-down. Due to the 20 bp overlapping regions in primers *dapA*-1 and *dapA*-2, the full-length homology fragment was generated via overlap PCR. This PCR product was electroporated into *Ec101* to achieve seamless knockout of *dapA*, resulting in strain *Ec102*. All primers used are listed in Table S1.

### 2.4. Phylogenetic Tree Construction

Multiple sequence alignment (MSA) of the target DNA sequences was performed using MEGA 11 software. All downloaded sequences were imported into the alignment window, and the Clustal W algorithm was selected for alignment with default parameters. After alignment, redundant terminal gaps and truncated sequence fragments caused by length discrepancies were manually trimmed, with only the conserved aligned regions retained. The final MSA results were saved in MEGA format for subsequent phylogenetic analysis. A phylogenetic tree was constructed based on the optimized MSA data using the Neighbor-Joining (NJ) method in MEGA 11. In the parameter setting interface, the Bootstrap method was employed as the validation approach, with the number of bootstrap replicates set to 1000 to evaluate the reliability of tree topology.

### 2.5. Conjugation

Cell density was determined by measuring the optical density at 600 nm (OD_600_). Based on OD_600_ values, 1 × 10^6^ cells (both donor and recipient) were harvested and washed twice with PBS. Donor cells were resuspended in 100 μL of LB medium supplemented with diaminopimelic acid (DAP), then transferred to a tube containing recipient cells to resuspend them. The mixture was spotted onto LB plates supplemented with DAP and incubated at 37 °C for 24 h. After incubation, cultures were scraped from the plates, resuspended in 500 μL of LB medium, and spread onto LB plates with chloramphenicol resistance for selection. Single colonies were picked and subjected to PCR verification using primers *oriT*-F/*oriT*-R, 27F/1492R and *dapA*-up/*dapA*-down, respectively.

Conjugation efficiency was calculated based on the PCR results using the following equation:

The conjugation successful ratio = the number of positive colonies/the number of colonies tested × 100%

### 2.6. Fluorescence Analysis and Quantification

To assess GFP expression, a defined quantity of bacterial cells was spotted onto a glass slide, covered with a coverslip, and briefly heated over an alcohol lamp for 5 s before air-drying. Fluorescence visualization was performed using a ZEISS Axiovert 5 microscope (Oberkochen, Germany) at 60× magnification with Openlab software (v3.8). For GFP quantification, fluorescence intensity was measured at an excitation wavelength of 488 nm and an emission wavelength of 507 nm. In this study, fluorescence intensity was calculated as arbitrary units (AU) using the GFP/OD_600_ ratio.

### 2.7. pAS104 Stability Test

*Pd103* harboring pAS104 was first inoculated into 10 mL of liquid LB medium to an initial OD_600_ of 0.1 and cultured in a 30 °C incubator. Samples were collected every 24 h and spread onto LB plates. Following colony growth, the same colony was tested on both LB plates and LB plus chloramphenicol plates. Colonies capable of growing on both plate types were considered positive. 100 colonies were tested per plate, and totally 300 colonies tested at each time point.

### 2.8. Inoculation of P. dechangensis NEAU-ST10-9^T^ or Pd103 with Maize Seeds

The maize seeds were purchased from Beijing Huanai Agricultural Development Co., Ltd., Beijing, China. Maize seeds of uniform size were selected and first rinsed with ultrapure water. Surface sterilization was performed by soaking seeds in 1% sodium hypochlorite solution for 2 min, followed by immersion in 75% ethanol solution for 5 min. Residual ethanol was then removed by rinsing the seeds with sterile ultrapure water three times. For the control group (CK group), five seeds were soaked in sterile ultrapure water for 6 h and placed in a Petri dish lined with two layers of filter paper, supplemented with 10 mL of ultrapure water. For the treatment group (inoculated with *P. dechangensis* NEAU-ST10-9^T^ or *Pd103*), maize seeds were soaked in a bacterial suspension (OD_600_ = 1) for 6 h and placed in a Petri dish lined with two layers of filter paper, supplemented with 10 mL of the same bacterial suspension (OD_600_ = 1). Each group comprised three biological replicates, with five maize seeds sown per plate (15 total seeds per group across three plates). All seeds were germinated in the dark for 6 days. Root lengths of different groups were measured using a ruler and subjected to statistical analysis.

### 2.9. Confocal Microscope Analysis

On the 6th day, fresh maize roots from the water-soaked control group and bacterial suspension-treated group were rinsed with ultrapure water. After blotting dry with lint-free paper, ~1 cm root segments were excised from each group and vertically embedded in 1% (*w*/*v*) sodium carboxymethyl cellulose (CMC) solution [14]. Sections (15 μm thick) were prepared using a clinical cryostat (LEICA CM1950 S; Leica, Wetzlar, Germany) and mounted on glass slides. Following air-drying, slides were stored at −80 °C (MDF-U74V; PHCbi, Tokyo, Japan) for 24 h prior to analysis.

Root sections were observed using a fast super-resolution laser scanning confocal microscope (LSM 900; ZEISS, Oberkochen, Germany) with excitation and emission wavelengths set at 488 nm and 528 nm, respectively. Each sample was analyzed in triplicate, and images were captured and processed using ZEN software (v3.8).

### 2.10. Statistical Analysis

The fluorescence of strains was calculated and statistically analyzed using one-way analysis of variance (ANOVA). Duncan’s multiple-range test was used when one-way ANOVA indicated a significant difference (*p* < 0.05). All statistical analyses were performed with Prism 11.0.1.

## 3. Results

### 3.1. Application of P. dechangensis NEAU-ST10-9^T^ in Promoting Maize Seedling Development

**Figure 1:**
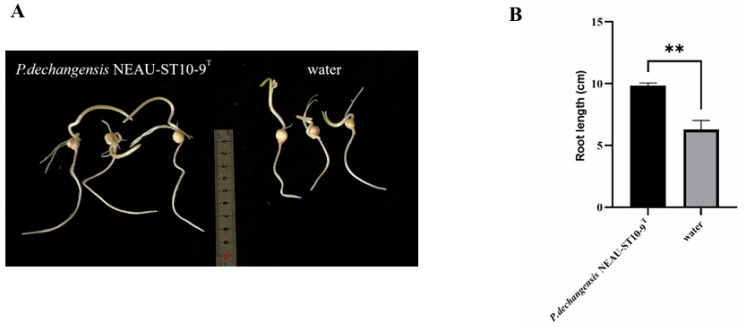
*P. dechangensis* NEAU-ST10-9^T^ could prompt maize seedling root development. (**A**) Images of maize roots under different treatments; (**B**) comparison of maize root lengths among different groups (** *p* < 0.01). Totally 15 samples in each group were tested.

Maize seeds were inoculated with a suspension of *P. dechangensis* NEAU-ST10-9^T^ and with water used as the control. Root lengths were compared between the two groups. The root length of the treatment group (*P. dechangensis* NEAU-ST10-9^T^) was 1.56 times that of the control group (water) (Figure 1A). Statistical analysis revealed a significant difference in root development between the treatment and control groups (*p* < 0.01), indicating that *P. dechangensis* NEAU-ST10-9^T^ promoted root growth of maize (Figure 1B).

### 3.2. Construction of Strain Ec102 and Evaluation of Its Conjugation Donor Capacity

**Figure 2:**
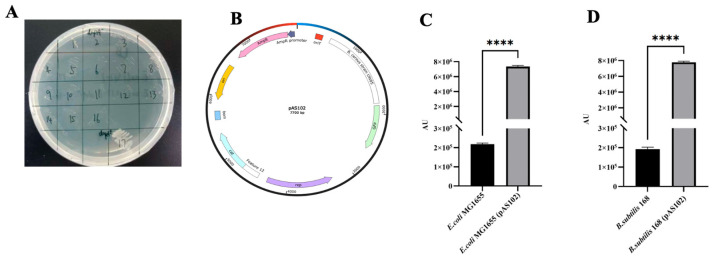
Conjugation system development. (**A**) *Ec102* growth test on LB plate with *E. coli* S17-1 as control (*dapA*− represented *Ec102*, and *dapA*+ represented *E. coli* S17-1; colonies 1 to 16 represented colonies from *Ec102,* while colony 17 represented *E.coli* S17-1). (**B**) Plasmid map of pAS102. (**C**) The fluorescence intensity comparison between *E. coli* MG1655 and *E. coli* MG1655 (pAS102) (**** *p* < 0.0001). (**D**) The fluorescence intensity comparison between *B. subtilis* 168 and *B. subtilis* 168 (pAS102) (**** *p* < 0.0001).

*DapA*, encoding 4-hydroxy-tetrahydrodipicolinate synthase, is an essential gene. Following its knockout, the strain becomes DAP-dependent [15]. In this study, *dapA* in *E. coli* S17-1 was deleted to generate strain *Ec102*. Strain *Ec102* was DAP-dependent and failed to grow on LB plates lacking DAP even after 7 days of incubation (Figure 2A). A plasmid pAS102 containing *oriT* was constructed from pNW33n-GFP and was capable of replicating in both *E. coli* and *Bacillus subtilis* (Figure 2B). To investigate whether *dapA* deletion affected conjugation efficiency, *Ec102* harboring pAS102 was conjugated with *Escherichia coli* MG1655 (*E. coli* MG1655) and *Bacillus subtilis* 168 (*B. subtilis* 168) separately. The fluorescence intensity of *E. coli* MG1655 (pAS102) was approximately 33.68 folds higher than that of *E. coli* MG1655 (without the plasmid) (Figure 2C), whereas the fluorescence intensity of *B. subtilis* 168 (pAS102) was approximately 40.33 folds higher than that of *B. subtilis* 168 (without the plasmid) (Figure 2D). In addition, *E. coli* MG1655 (pAS102) and *B. subtilis* 168 (pAS102) both displayed significantly enhanced fluorescence intensity under blue light irradiation relative to their respective parental strains (Figure S1).

### 3.3. Development of a Conjugation System for P. dechangensis NEAU-ST10-9^T^

**Figure 3:**
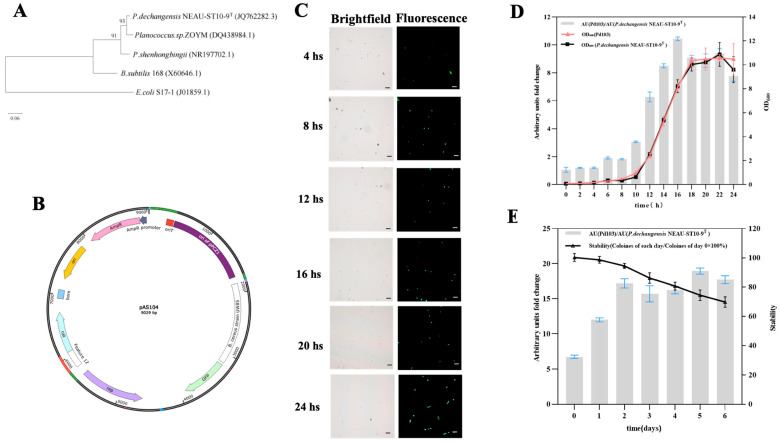
GFP overexpression in *P. dechangensis* NEAU-ST10-9^T^. (**A**) Phylogenetic tree showing the close genetic relationship between *P. dechangensis* NEAU-ST10-9^T^ and *Planococcus* sp. ZOYM. (**B**) Schematic diagram of plasmid pAS104. (**C**) Microscopic fluorescence analysis of *Pd103*. (**D**) Fluorescence intensity of *Pd103* correlated with its growth phase. (**E**) Plasmid stability and fluorescence intensity dynamics of *Pd103* over a six-day incubation period. Arbitrary unit (AU) fold change is defined as the ratio of AU (*Pd103*) to AU (*P. dechangensis* NEAU-ST10-9^T^). Plasmid stability was calculated using the formula: (number of positive colonies on each day/number of positive colonies on day 0) × 100%.

Firstly, the chloramphenicol tolerance of *P. dechangensis* NEAU-ST10-9^T^ was tested. The strain failed to grow on LB agar plates containing 34 μg/mL chloramphenicol after 48 h of cultivation, so 34 μg/mL chloramphenicol was used for selection purposes. *P. dechangensis* NEAU-ST10-9^T^ was phylogenetically distant from both *E. coli* S17-1 and *Bacillus subtilis* 168, but closely related to *Planococcus* sp. ZOYM according to 16s rDNA analysis (Figure 3A). *Planococcus* sp. ZOYM harbors a natural plasmid, pCZ1, whose sequence has been deposited in the NCBI database (https://www.ncbi.nlm.nih.gov/nuccore/NC_013539.1, 4 April 2026). Closely phylogenetically related bacterial strains may exhibit tolerance to plasmids bearing the same replicon. Thus, the minimal replicon of pCZ1 was synthesized, cloned into pAS102, and finally used to construct plasmid pAS104 (Figure 3B). pAS104 was electroporated into *Ec102*, and the transformed cells were plated on LB agar supplemented with DAP and chloramphenicol for cultivation. Subsequently, *Ec102* harboring pAS104 was conjugated with *P. dechangensis* NEAU-ST10-9^T^ to generate strain *Pd103*, with a conjugation success rate of 100% (Figures S2 and S3).

A single colony of *Pd103* was picked and cultured overnight in liquid LB medium supplemented with chloramphenicol. The culture was then inoculated into 50 mL of liquid LB medium supplemented with chloramphenicol to an initial OD_600_ of 0.1. The same procedure was performed for *P. dechangensis* NEAU-ST10-9^T^. Samples were collected every 4 h and subsequently analyzed. Fluorescence intensity increased over the cultivation period, reaching a maximum at 16 h based on the brightness of cells observed under a fluorescence microscope (Figure 3C). Subsequently, it decreased at 20 h (Figure 3C).

Fluorescence was also analyzed every two hours by calculating the ratio of AU (*Pd103*) to AU (*P. dechangensis* NEAU-ST10-9^T^). *Pd103* showed the strongest fluorescence after 16 h of culturing, with a fluorescence intensity approximately 10-fold higher than that of *P. dechangensis* NEAU-ST10-9^T^ (Figure 3D). This result was consistent with the fluorescence observations under the microscope. Based on the growth curve, *Pd103* entered the stationary phase after 18 h of culturing, indicating that fluorescence intensity in *Pd103* correlated with its growth during the log phase (Figure 3D). After *Pd103* entered the stationary phase, fluorescence intensity gradually decreased.

### 3.4. Evaluation of pAS104 Stability in Strain Pd103

When high numbers of microorganisms were inoculated at plant roots, microbial-plant interactions can be observed as early as 24 h post-inoculation [16,17]. Based on this, vectors carrying GFP should remain stable even in the absence of selection pressure (e.g., antibiotics). In this study, the stability of pAS104 in *Pd103* was evaluated by determining the proportion of positive colonies on LB plates supplemented with chloramphenicol every 24 h. As Figure 3E shows, it was 100% after 24 h and remained at 70% even after six days (Figure 3E). The fluorescence intensity of *Pd103* relative to *P. dechangensis* NEAU-ST10-9^T^ was also monitored, which remained above 17-fold following six days of incubation (Figure 3E). These data indicated that pAS104 remained highly stable in *Pd103*, suggesting that the fluorescence labeling system established in this study can be further applied to track interactions between *Pd103* and plants.

### 3.5. Investigation of Pd103 Colonization in Maize Roots

**Figure 4:**
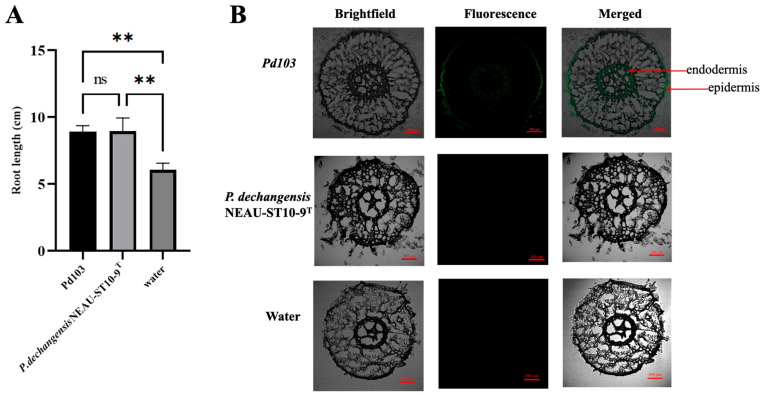
Effects of *Pd103* on maize root growth and its colonization in maize roots. (**A**) Comparison of maize root lengths among different groups (** *p* < 0.01, ns stands no significant difference, *n* = 15 per group). (**B**) Localization of *Pd103* in maize roots. No GFP signal was observed in the *P. dechangensis* NEAU-ST10-9^T^ or water control group.

To investigate the absorption and distribution characteristics of *Pd103* in maize roots, maize seeds were inoculated with *Pd103* bacterial suspension, while the control group received sterile water inoculation. To assess the autofluorescence of *P. dechangensis* NEAU-ST10-9^T^, maize seeds were also inoculated with this strain. As shown in Figure 4A, both *Pd103* and *P. dechangensis* NEAU-ST10-9^T^ could prompt maize root development. As shown in Figure 4B, no fluorescent signals were detected in the root cross-sections of the water group as well as of *P. dechangensis* NEAU-ST10-9^T^ group, confirming the absence of background interference in the experimental system. *Pd103* colonization was predominantly observed in the epidermis and endodermis of maize roots (Figure 4B and Figure S4). According to fluorescence-based observations, *Pd103* preferentially accumulated in structural tissues involved in nutrient transport and mechanical support.

## 4. Discussion

Owing to their unique cell wall structures and complex genetic backgrounds, *Planococcus* strains are recalcitrant to conventional genetic manipulation techniques such as transformation and electroporation, which has long impeded progress in the molecular biological research of this genus. In particular, reliable fluorescent tracing tools for investigating host–microbe interactions have been lacking. Herein, we innovatively applied conjugation using an optimized auxotrophic donor strain to efficiently deliver a GFP-expressing plasmid into *P. dechangensis* NEAU-ST10-9^T^. Following antibiotic selection and fluorescence verification, the labeled strain exhibited stable and uniform GFP expression without significant fluorescence attenuation, thereby overcoming the technical bottleneck of genetic labeling in this genus. The establishment of this labeling system not only provided a critical tool for the successful implementation of root colonization assays in the present study but also filled the gap in fluorescent tracing technology for *Planococcus*, laying an important methodological foundation for future research on the endophytic traits, host interactions, and environmental adaptability of this genus. Except for GFP, there are other fluorescent proteins available, including RFP (a red fluorescent protein), YFP (a yellow fluorescent protein) and CFP (a cyan fluorescent protein) [18,19]. These fluorescent proteins exhibit mutual compatibility and represent promising molecular tools for the in situ tracking of *P. dechangensis* NEAU-ST10-9^T^, thereby facilitating investigations into microbial community interactions and microbe–plant interactions.

In the methodological design of this study, the selection of the auxotrophic donor strain and screening of the pCZ1 replicon were critical for ensuring conjugation efficiency and labeling stability, although certain design limitations were also identified. For the donor strain, we developed a diaminopimelic acid (DAP)-auxotrophic *E. coli* strain as the conjugal donor. Its key advantage is the ability to effectively distinguish donor cells from *P. dechangensis* NEAU-ST10-9^T^ via medium-based selection: simply omitting DAP from the selection medium inhibits the growth of the donor strain, avoiding interference with subsequent experimental results and simplifying the screening process for labeled recombinants. This strategy relies on the inherent ability of *P. dechangensis* NEAU-ST10-9^T^ to synthesize DAP autonomously, ensuring the normal growth of the target strain on selective media and greatly improving screening efficiency. Plasmid stability is a critical attribute for both industrially engineered microbial strains and plant-associated microorganisms. Since heterologous protein expression is readily achieved via plasmids, plasmid stability directly modulates the yields of target protein expression. To mitigate this limitation, protein-encoding genes are integrated into bacterial chromosomes. However, this strategy often results in suboptimal protein expression levels due to the low copy number of chromosomally integrated genes. Cloning replicons from native bacterial plasmids represents a well-validated approach to enhance the stability of engineered plasmids [20] (Sawisit et al., 2015). Regarding the replicon, pCZ1 is derived from a native plasmid harbored by *Planococcus* sp. ZOYM. Experimental results confirmed that, due to the close phylogenetic relationship between the two strains, pAS104 carrying the pCZ1 replicon is relatively stable in *P. dechangensis* NEAU-ST10-9^T^, supporting the sustained expression of GFP. However, an objective limitation was observed during long-term culture: under non-selective conditions without antibiotics, 30% of the colonies failed to grow on LB plates containing chloramphenicol after six days of culture, indicating plasmid loss. This instability may be influenced by the metabolic status of the host strain. Future studies could further improve the long-term stability of the labeling system by optimizing replicon sequences or introducing plasmid stability elements.

Numerous PGPR strains (e.g., *Streptomyces* spp. and *Bacillus velezensis*) improve plant growth by indirectly modulating the rhizosphere microenvironment and regulating host defense responses [21,22]. However, reports on the plant growth-promoting traits of *Planococcus* remain limited. Following the development of the fluorescence system, maize seeds were inoculated with *Pd103* to investigate how it promoted maize root development. Laser confocal microscopy results showed that *Pd103* colonized at the roots. This colonization pattern is similar to that of closely related endophytic genera, suggesting that *Pd103* may adopt a typical colonization strategy utilized by beneficial plant growth-promoting bacteria.

Based on the current data, the underlying growth-promoting mechanism of *Pd103* in maize remains unclear. IAA is a key phytohormone that regulates plant growth. In our experiments, inoculation with *P. dechangensis* NEAU-ST10-9^T^ significantly increased the root length of maize roots compared with the control group. We therefore hypothesize that this strain may secrete IAA, which in turn promotes plant growth. However, the current experimental evidence is insufficient to support this conclusion. The future work will focus on elucidating the colonization mechanisms and perform multi-omics analyses on *P. dechangensis* NEAU-ST10-9^T^ to determine whether it can synthesize IAA and whether the produced IAA affects the expression of phytohormone signaling-related genes in the maize, thereby clarifying whether the growth-promoting mechanism of this strain is IAA-dependent. In addition, we only evaluated the effects of *P. dechangensis* NEAU-ST10-9^T^ on maize root development. However, given that this strain harbors multiple salt tolerance-associated genes [12], a key direction for future research will be to investigate its functional role in maize under salt stress conditions.

## 5. Conclusions

This is the first study to confirm that *P. dechangensis* NEAU-ST10-9^T^ can promote root development in maize seedlings. Furthermore, we reported the first establishment of an effective fluorescence tracking system to monitor *Pd103* following coculture with maize seeds and proved that the strain colonized the epidermis and endodermis of roots. This work thus provides a valuable model for investigating rhizosphere microorganism–plant interactions. Moreover, this system provides a robust platform for the exploration of additional PGPR resources, which in turn lays a solid foundation for the development of highly efficient biofertilizers.

## 6. Patents

This work has been included in a patent with application number 202511480236.4.

## Figures and Tables

**Table 1:** Strains and plasmids used in this study.

Strains or Plasmids	Relevant Genotype	Source or Reference
Strains		
*E. coli* S17-1	Tp^R^ Sm^R^ *recA*, *thi*, *pro*, *hsdR*-M^+^ RP4-2(Km::Tn7 Tc::Mu-1)	Lab collection
*P. dechangensis* NEAU-ST10-9^T^		CGMCC [12]
*AS010*	*E.coli ATCC8739,adhE::cat-sacB*	Lab collection
*Ec101*	*E.coli* S17-1, *dapA*::*cat-sacB*	This work
*Ec102*	*E.coli* S17-1, Δ *dapA*	This work
*Pd103*	*P.dechangensis* NEAU-ST10-9^T^ with pAS104	This work
Plasmids		
pNW33n-GFP	*gfp* gene with lac promoter was cloned into pNW33n.	Lab collection
pAS101	*oriT* of RP4 was synthesized and cloned into pUC18.	This work
pAS102	*oriT* of RP4 was cloned into pNW33n-GFP.	This work
pAS103	The Minimal Replicon of pCZ1 was synthesized and cloned into pUC18.	This work
pAS104	The Minimal Replicon of pCZ1 was cloned into pAS102.	This work

## Data Availability

The original contributions presented in this study are included in the article/Supplementary Material. Further inquiries can be directed to the corresponding authors.

## Supplementary Materials

The following supporting information can be downloaded at: https://www.mdpi.com/article/10.3390/microorganisms14051139/s1, Table S1: Primers used in this study. Figure S1: Comparison of fluorescence intensity under blue light irradiation. Figure S2: PCR amplification and DNA gel electrophoresis analysis of transconjugant colonies from conjugation between *P. dechangensis* NEAU-ST10-9^T^ and *Ec*102. Figure S3: A scheme for strain *Pd*103 development. Figure S4: *Pd*103-treated maize seedlings observed via inverted fluorescence microscope at 1000× total magnification (10× eyepiece, 100× objective lens).
