# Supplementary material for: Planococcus dechangensis NEAU-ST10-9T Promotes Maize Seedling Root Development: Evidence from Effective Fluorescence Tracking
Source: Microorganisms. 2026 May 17;14(5):1139. doi: 10.3390/microorganisms14051139 (PMC13209848; doi:10.3390/microorganisms14051139)
Supplement: Supplementary file 1 [file microorganisms-14-01139-s001.zip › microorganisms-4229553-figures.pptx]

## Slide 1
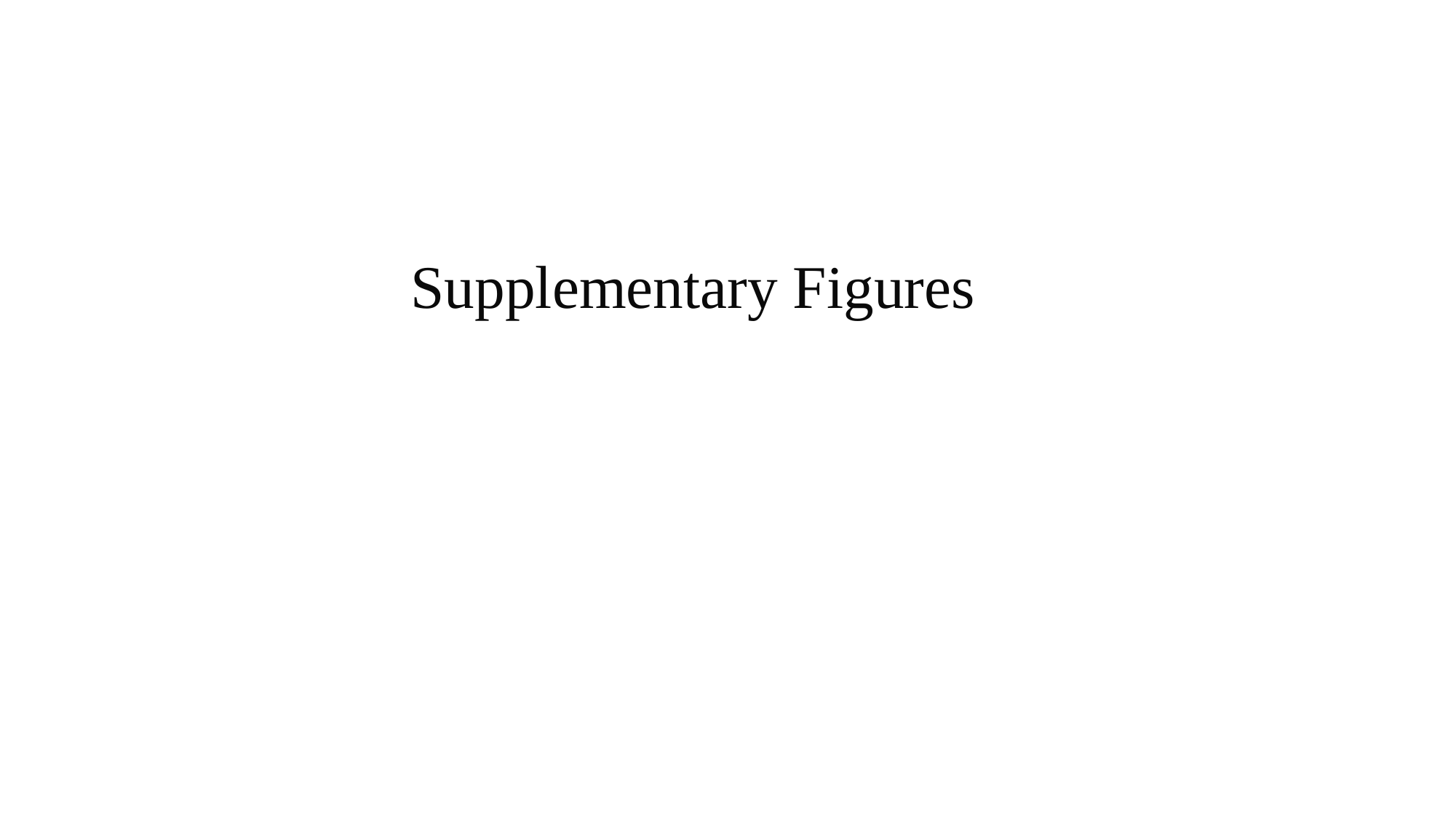

Supplementary Figures

## Slide 2
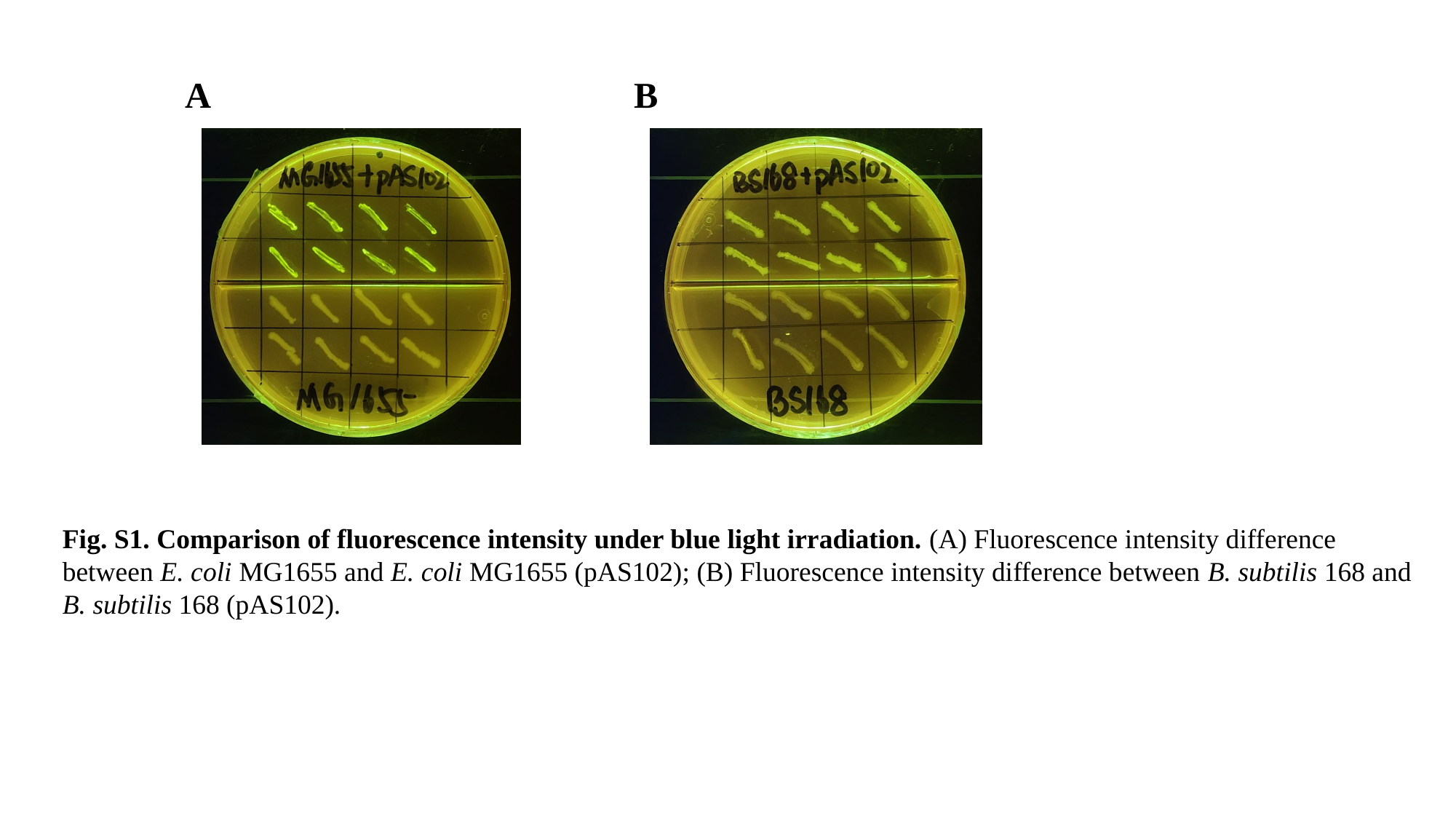

B
A
Fig. S1. Comparison of fluorescence intensity under blue light irradiation. (A) Fluorescence intensity difference between E. coli MG1655 and E. coli MG1655 (pAS102); (B) Fluorescence intensity difference between B. subtilis 168 and B. subtilis 168 (pAS102).

## Slide 3
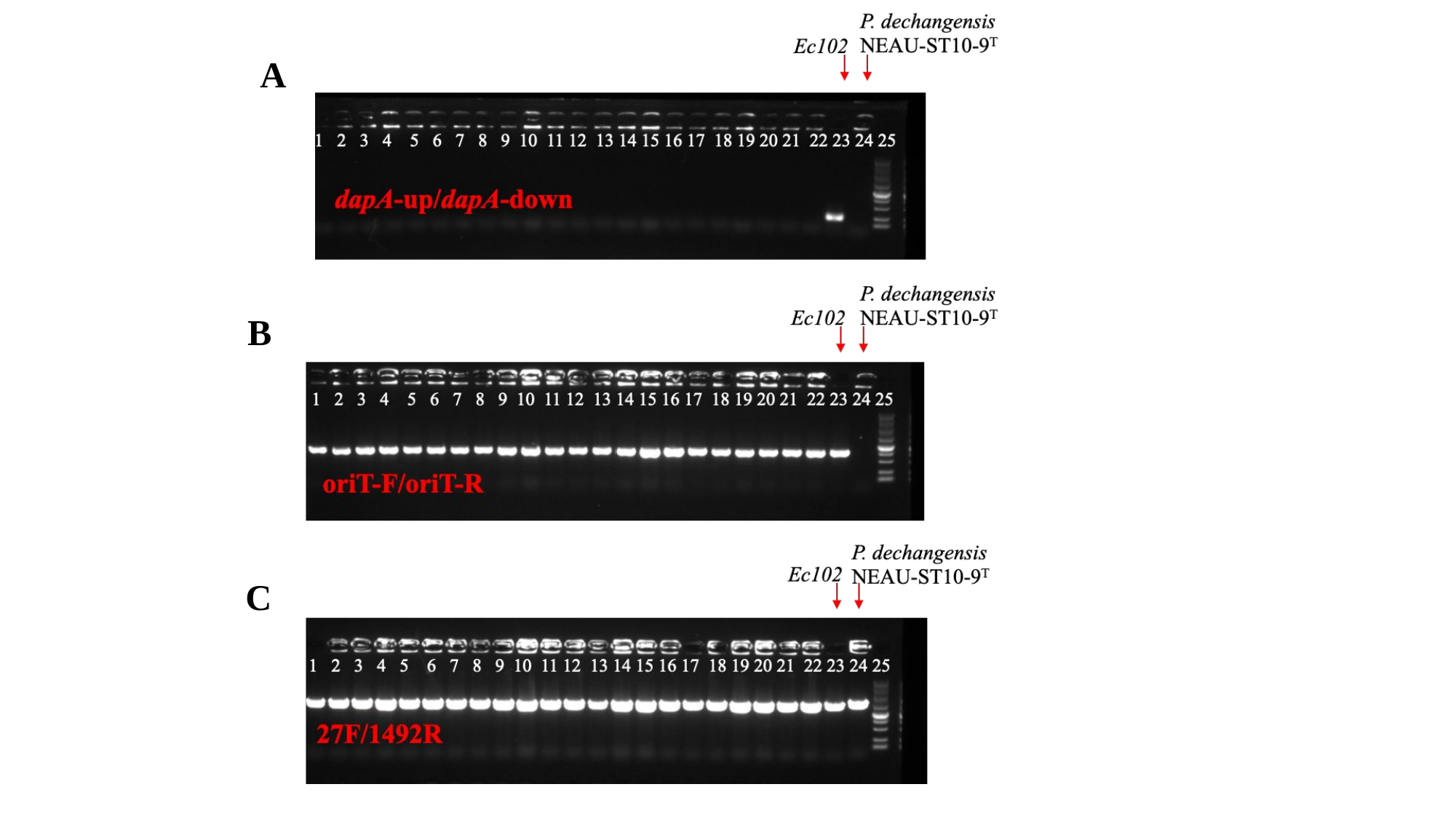

A
B
C

## Slide 4
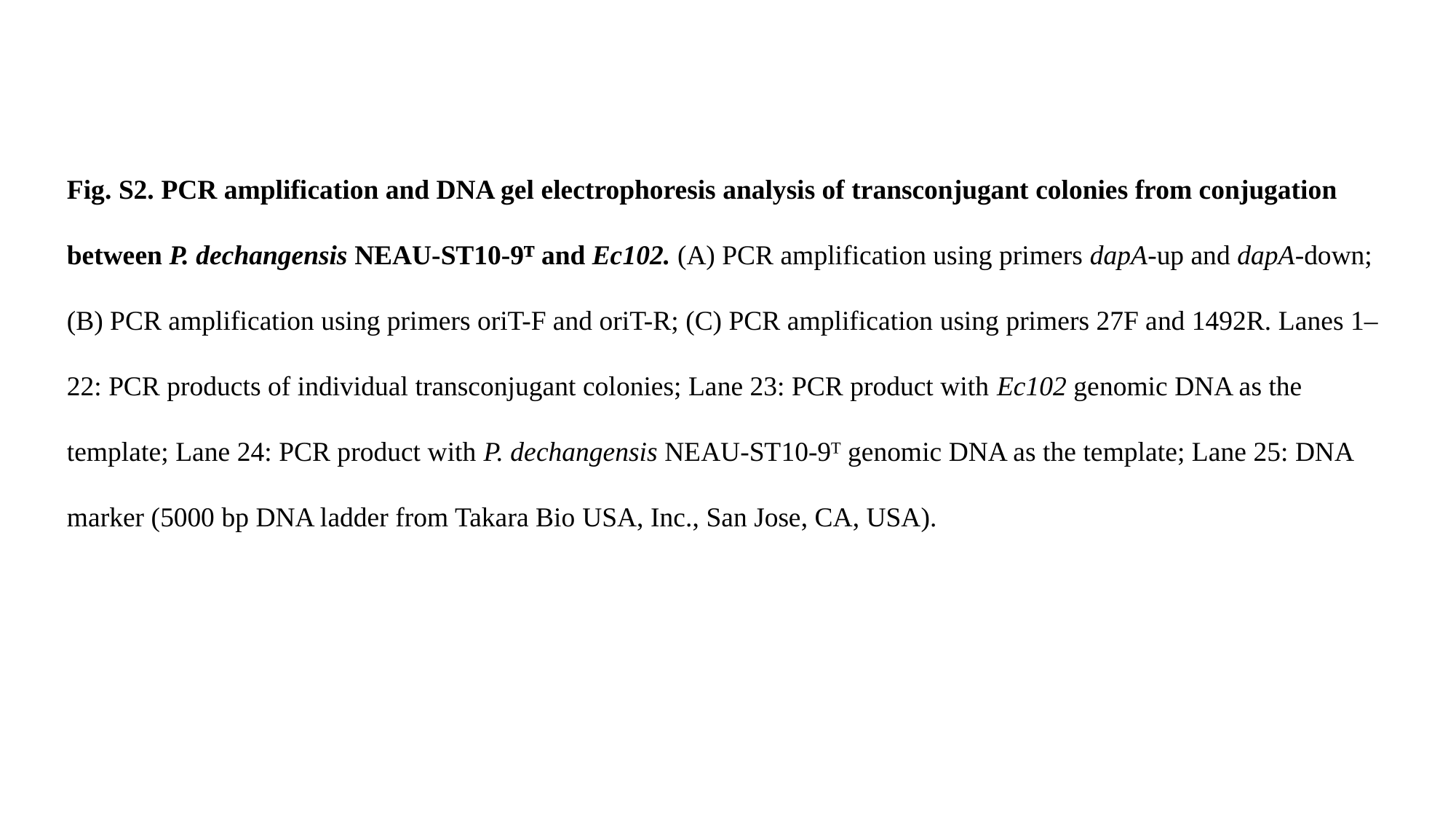

Fig. S2. PCR amplification and DNA gel electrophoresis analysis of transconjugant colonies from conjugation between P. dechangensis NEAU-ST10-9ᵀ and Ec102. (A) PCR amplification using primers dapA-up and dapA-down; (B) PCR amplification using primers oriT-F and oriT-R; (C) PCR amplification using primers 27F and 1492R. Lanes 1–22: PCR products of individual transconjugant colonies; Lane 23: PCR product with Ec102 genomic DNA as the template; Lane 24: PCR product with P. dechangensis NEAU-ST10-9ᵀ genomic DNA as the template; Lane 25: DNA marker (5000 bp DNA ladder from Takara Bio USA, Inc., San Jose, CA, USA).

## Slide 5
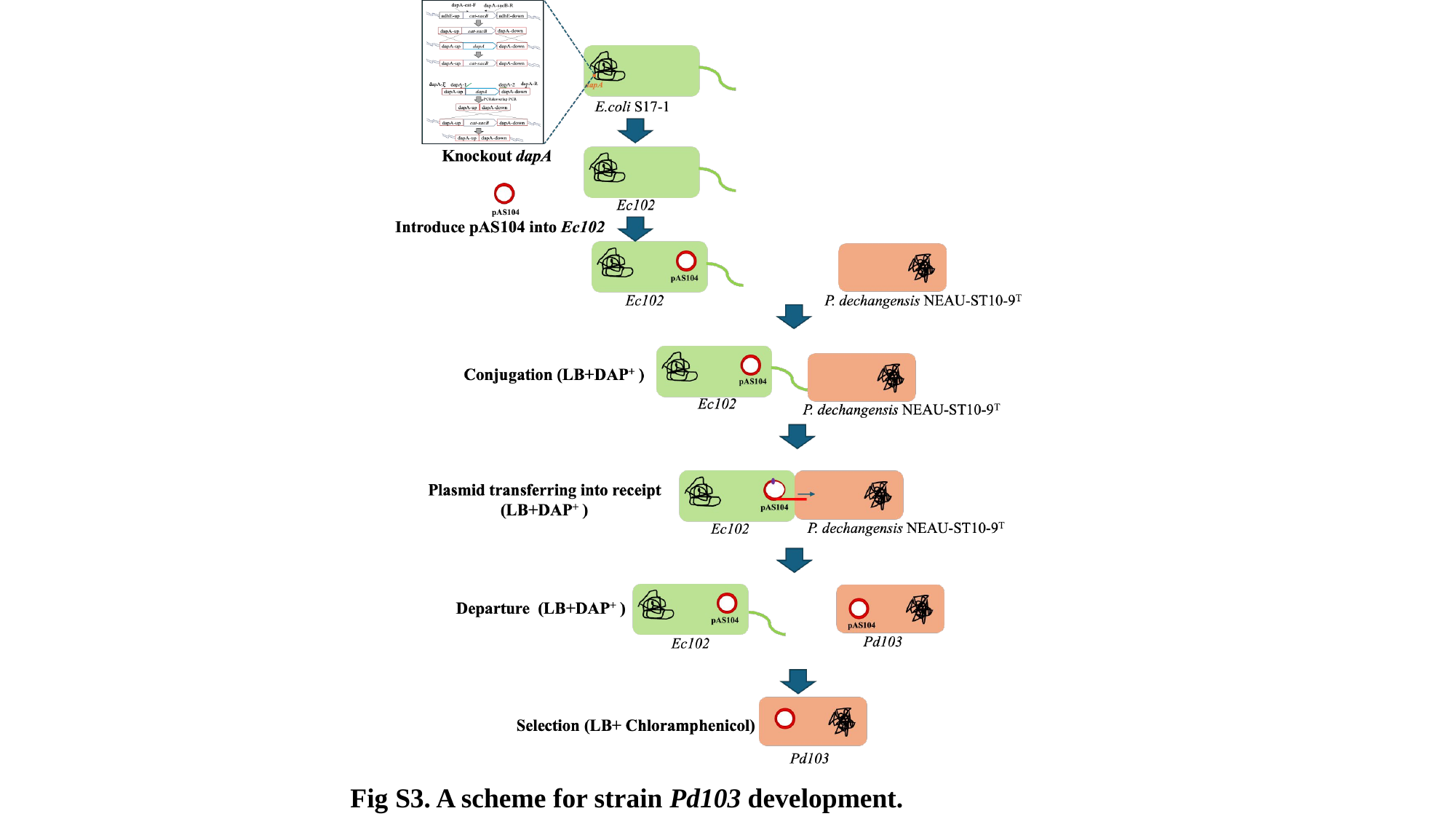

Fig S3. A scheme for strain Pd103 development.

## Slide 6
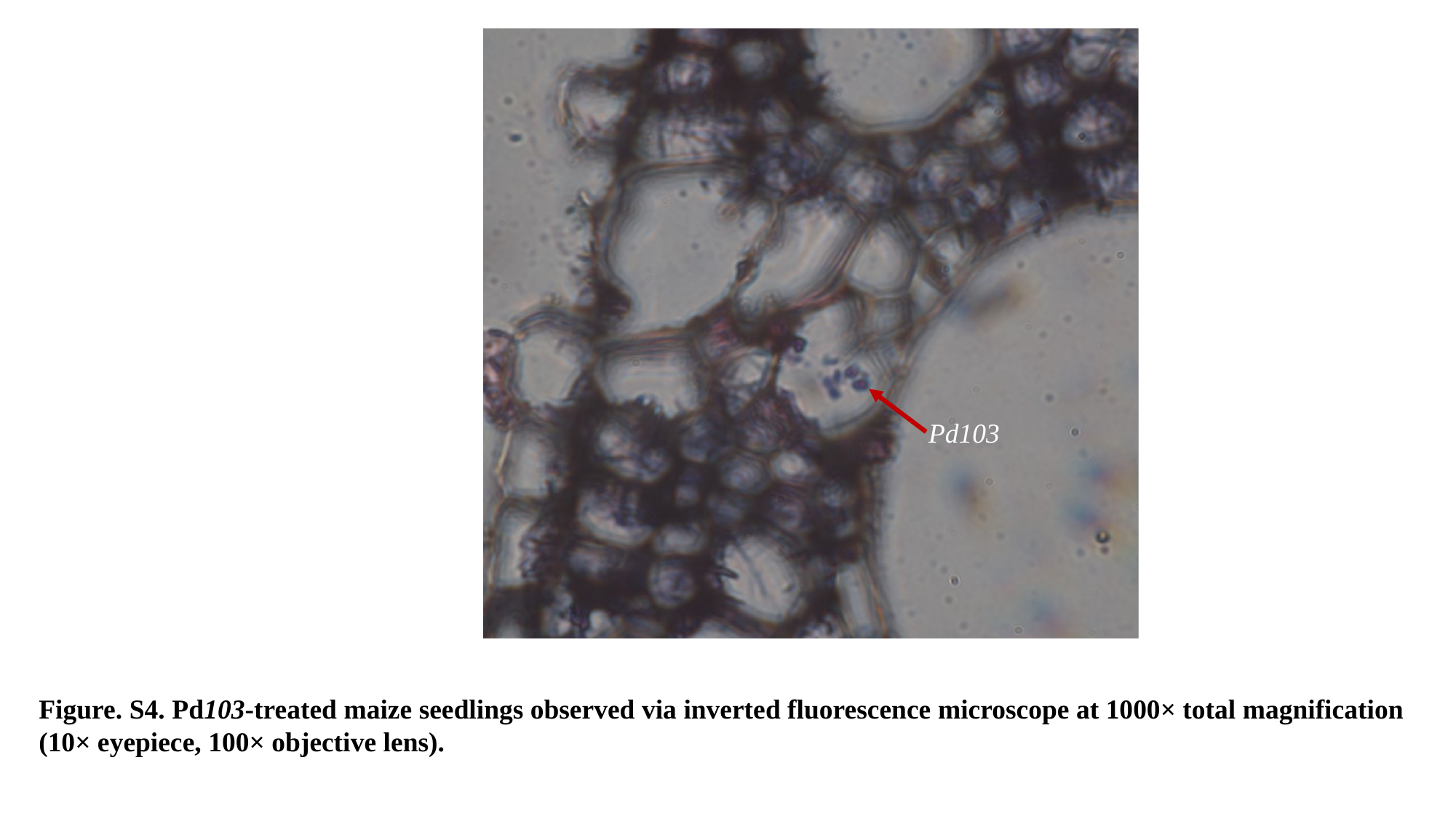

Pd103
Figure. S4. Pd103-treated maize seedlings observed via inverted fluorescence microscope at 1000× total magnification (10× eyepiece, 100× objective lens).
