# Supplementary material for: Planococcus dechangensis NEAU-ST10-9T Promotes Maize Seedling Root Development: Evidence from Effective Fluorescence Tracking
Source: Microorganisms. 2026 May 17;14(5):1139. doi: 10.3390/microorganisms14051139 (PMC13209848; doi:10.3390/microorganisms14051139)
Supplement: Supplementary file 1 [file microorganisms-14-01139-s001.zip › microorganisms-4229553-table.pdf]

Table S1. Primers used in this study

Primer name	Oligonucleotide sequence (5′–3′)
dapA-cat-F	TTGCCGGGACCGGCGCTAACGCTACTGCGGAAGCCATTAGCCTGACG CAGTGTGACGGAAGATCACTTCGCA
dapA-sacB-R	CAGTTTGCACATCTGGGCCATATCACGCGCTGCGACGTTAGCCGTAA CGGGCGTTTTTATTTGTTAACTGTTAATTGTCCT
dapA-up	GTATTGTCGCGATTGTTACTCCGATGG
dapA-1	GGCTAATGGCTTCCGCAGTA
dapA-2	TACTGCGGAAGCCATTAGCCAGCGCGTGATATGGCCCAG
dapA-down	CCGGCATGCTTAAGCGCC
oriT-F	TTGAGTCAGCTAGGAGGTGACTGAAG
oriT-R	GCCAATAAGGCCTTTCTAGATTTCTAG
pNW33N-F	TCTAGAAAGGCCTTATTGGCTTCACAATCAGAGAGAACGGG
pNW33N-R	TCACCTCCTAGCTGACTCAACCGCTCTAGAACTAGTGGATC
rep-pPCZ1-F	CTGGGGCCAGATGGTAAGATTACGCTTTTAGTTCTTTTTCAC
rep-pPCZ1-R	CAGTGGAACGAAAACTCACGTCAACTTCTATCTTCTAACCAATTATA AAATTC
pNW33N-origin-cloning-F	CGTGAGTTTTCGTTCCACTG
pNW33N-origin cloning-R	CTTACCATCTGGCCCCAG
27F	AGAGTTTGATCCTGGCTCAG
1492R	GGTTACCTTGTTACGACTT
